# Preventing knee injuries in adolescent female football players – design of a cluster randomized controlled trial [NCT00894595]

**DOI:** 10.1186/1471-2474-10-75

**Published:** 2009-06-23

**Authors:** Martin Hägglund, Markus Waldén, Isam Atroshi

**Affiliations:** 1 Department of Medical and Health Sciences, Linköping University, 581 83 Linköping, Sweden; 2 Department of Orthopedics, Hässleholm and Kristianstad Hospitals, Box 351, 281 25 Hässleholm, Sweden; 3 Department of Clinical Sciences, Lund University, Box 117, 221 00 Lund, Sweden

## Abstract

**Background:**

Knee injuries in football are common regardless of age, gender or playing level, but adolescent females seem to have the highest risk. The consequences after severe knee injury, for example anterior cruciate ligament (ACL) injury, are well-known, but less is known about knee injury prevention. We have designed a cluster randomized controlled trial (RCT) to evaluate the effect of a warm-up program aimed at preventing acute knee injury in adolescent female football.

**Methods:**

In this cluster randomized trial 516 teams (309 clusters) in eight regional football districts in Sweden with female players aged 13–17 years were randomized into an intervention group (260 teams) or a control group (256 teams). The teams in the intervention group were instructed to do a structured warm-up program at two training sessions per week throughout the 2009 competitive season (April to October) and those in the control group were informed to train and play as usual. Sixty-eight sports physical therapists are assigned to the clubs to assist both groups in data collection and to examine the players' acute knee injuries during the study period. Three different forms are used in the trial: (1) baseline player data form collected at the start of the trial, (2) computer-based registration form collected every month, on which one of the coaches/team leaders documents individual player exposure, and (3) injury report form on which the study therapists report acute knee injuries resulting in time loss from training or match play. The primary outcome is the incidence of ACL injury and the secondary outcomes are the incidence of any acute knee injury (except contusion) and incidence of severe knee injury (defined as injury resulting in absence of more than 4 weeks). Outcome measures are assessed after the end of the 2009 season.

**Discussion:**

Prevention of knee injury is beneficial for players, clubs, insurance companies, and society. If the warm-up program is proven to be effective in reducing the incidence of knee injury, it can have a major impact by reducing the future knee injury burden in female football as well as the negative long-term disabilities associated with knee injury.

**Trial registration:**

NCT00894595

## Background

Football is the most popular sport worldwide. Unfortunately, football-related knee injuries are common and constitute a serious problem regardless of the playing level. The injury that draws most attention is the anterior cruciate ligament (ACL) injury. This injury usually causes long absence from football and may even force some players to give up their career [1]. ACL injury is also associated with an increased risk of new knee injury [2,3], as well as long-term medical disability related to osteoarthritis [4,5].

There are, however, only few investigated risk factors for ACL injury in football. First, many studies have shown a higher incidence among female than male players [6]. Second, low age is a risk factor for female players and the risk seems to be highest during the late pubertal or first postpubertal years [7]. Third, match play is associated with a considerably higher risk of ACL injury compared to training [8-11]. Several other potential risk factors, for example anatomical and biomechanical factors, have repeatedly been discussed in the literature, but lack scientific evidence [12]. Recently, it has been speculated that a high match frequency as well as match play at senior level could be a risk factor among female adolescent football players [1], but evidence for such a relationship is currently lacking.

Several studies have been conducted with the aim of preventing serious knee injuries and/or ACL injuries in female football [13-20]. However, only two of these studies have shown that the risk of ACL injury can be reduced using a comprehensive warm-up program [13,16]. In one study, the injury risk may actually have been increased in the group allocated to balance board training [20]. Unfortunately, many of the studies have serious methodological limitations, such as having few included participants or high drop-out rates resulting in low statistical power [14,15,17,20]. Furthermore, the studies commonly do not account for true exposure (playing time) resulting in a less accurate estimation of the actual injury risk [13-17]. Finally, another well-known problem is a poor compliance with the intervention [19], probably due to very ambitious programs taking too much time from the ordinary football training.

A structured warm-up program specifically developed for youth and adolescent team sports has been recently introduced in Sweden (Knäkontroll, SISU Idrottsböcker^©^, Sweden, 2005). This commercially available program contains six exercises focusing on neuromuscular knee control and core stability and is intended to be administrated by coaches. The effectiveness of the program in reducing the incidence of knee injury has, however, not been evaluated. The purpose of the present study is to conduct a cluster randomized controlled trial (RCT) evaluating the effectiveness of the program in adolescent female football players. Our hypothesis is that the structured warm-up program reduces the incidence of ACL injury as well as the incidence of any acute knee injury (except contusion) and severe knee injury (defined as injury resulting in absence of more than 4 weeks).

## Methods

### Study design and definitions

The study is a two-armed RCT designed in accordance with the CONSORT statement guidelines [21]. The definitions follow the international guidelines for football injury research published recently (Table 1) [22,23]. The study was registered in ClinicalTrials.gov at the start of the study .

**Table 1:** Operational definitions used in the study.

**Term**	**Definition**
Activity	Scheduled training session or match carried out with the player's own or another team.
Training session	Team training that involved physical activity under the supervision of the team coach.
Match	Friendly or competitive match against another team.
Acute knee injury	Injury to the knee joint with sudden onset and known cause, excluding contusions, leading to a player being unable to fully participate in future training or match play (i.e. time loss injury).
Sprain	Acute injury to ligament or joint capsule.
Meniscus lesion	Acute injury to the medial or lateral meniscus (verified by MRI or surgery).
Cartilage lesion	Acute injury to the articular cartilage (verified by MRI or surgery).
Bone marrow lesion	Acute injury to the bone marrow including occult fractures (verified by MRI).
Fracture	Acute injury to the bone (verified by plain X-ray).
Dislocation	Acute partial or complete dislocation of the patellofemoral joint or the knee joint.
Re-injury	Injury of the same type and to the same site as a previous injury the player had sustained in her career.
Early recurrent injury	Re-injury within two months of return to full participation after the index injury.
Late recurrent injury	Re-injury more than two months after return to full participation from the index injury.
Injury severity	Time elapsed from injury to full participation (days).
Minimal injury	Injury causing 1–3 days absence from training and match play.
Mild injury	Injury causing 4–7 days absence from training and match play.
Moderate injury	Injury causing 8–28 days absence from training and match play.
Severe injury	Injury causing >28 days absence from training and match play.
Injury mechanism	
Contact injury	Injury resulting from contact with another player or object, but not the surface.
Non-contact injury	Injury not resulting from contact with another player or object.
Injury incidence	Number of injuries per 1000 player-hours.

MRI: Magnetic Resonance Imaging

### Club recruitment

Eight regional districts of the Swedish Football Association (FA) located in the south and middle of Sweden were invited to participate in the study being carried out during the 2009 campaign. Leading officials from the selected districts were contacted by telephone in August 2008 and all agreed to support club recruitment to the study. Only female players aged 13–17 years (born 1992 to 1996) were eligible for inclusion in the study. Clubs were informed about the trial by advertisement on the websites of each regional district and through invitational e-mails and letters in December 2008. Club enrolment registries for the 2009 season were obtained for the U-14 to U-18 series from all districts in February 2009 and the coaches of all eligible teams were contacted by the authors (MH and MW). Some coaches of teams belonging to the senior league systems responded to the invitation and these teams were included if the squads consisted mainly of adolescent girls in the age groups eligible for study. Teams that did not respond to the invitation or declined participation, those that had less than two scheduled training sessions per week, and those already using the structured warm-up program or a similar preventive program were not included in the study.

### Randomization

**Figure 1:**
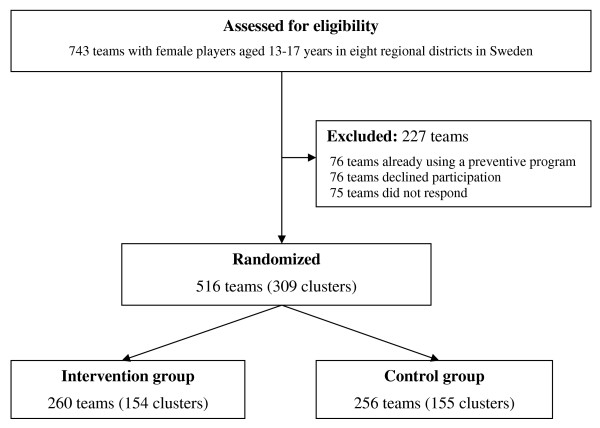
**Flow of teams and clusters (all teams from the same club are assigned to the same cluster) through the trial**.

All clubs (516 teams) that agreed to participate were randomized into an intervention or control group (Figure 1). All teams from the same club were assigned to the same group. The computer-generated cluster randomization was performed by one of the authors (IA) who is not involved in the intervention.

### Study therapist education

Physical therapists were recruited to the study to educate the coaches and assist the clubs with data collection. The study therapists were members of the Swedish Society of Football Physical Therapists or the Swedish Society of Sports Medicine. A 3-hour educational meeting for the study therapists was held by the authors (MH and MW) in November 2008. The meeting consisted of a presentation of the theoretical background to knee injury epidemiology in female adolescents and description of the study design followed by a practical education in the program given by an experienced supervisor. The participating therapists also received a specific manual describing the study methodology in detail as well as the tasks associated with the study. A few therapists, who were not yet recruited at the time of the educational meeting, received the same information on a later occasion before the start of the study. A total of 68 therapists accepted to participate in the trial.

### Coach education

All randomized teams were invited to regional instructional meetings held by the authors (MH and MW) before the start of the trial. In the intervention group, one coach or team leader from each club together with one player ("team captain") took part in a 3-hour meeting. Knee injury epidemiology in female adolescents, the study design and the data collection procedures were presented. This theoretical session was followed by a practical education in the structured warm-up program given by the study therapists. Each coach/team leader received a CD and a leaflet describing the warm-up exercises. In addition, they received a study manual describing the methodology, operational definitions and data collection procedures. The manual contains several examples of how to fill in the study forms. Coaches or team leaders that could not attend the educational meetings were sent the same theoretical session as a slide show presentation and received the practical education by their study therapists before the start of the trial.

Coaches of clubs assigned to the control group received a one-hour lecture about study background and methodology as well as the study manual but were informed to train and play as usual throughout the season. They were also informed that if the study shows that the warm-up program is successful in preventing knee injuries they will be offered the same practical education of the exercises before the start of the 2010 season.

### Intervention

The structured warm-up program (Knäkontroll, SISU Idrottsböcker^©^, Sweden, 2005) was developed for youth and adolescent team sports and is intended to be administrated by coaches. It was designed by experienced physical therapists involved in the medical organisations of the Swedish Football Association (FA) in collaboration with the Swedish Handball Federation, the Swedish Basketball Federation and the Swedish Floorball Federation. It contains six exercises focusing on neuromuscular knee control and core stability: (1) one-legged knee squat, (2) pelvic lift, (3) two-legged knee squat, (4) the bench, (5) the lunge, and (6) jump/landing (Table 2). Each exercise is subdivided into four steps of progressing difficulty (A to D) and each association/federation adjusted the progression of the exercises to be sport-specific. In addition, a pair-exercise was developed for intermittent use with the aim of making the training more varying and fun (Table 2). The structured warm-up program is commercially available on a CD with a corresponding leaflet describing the execution and purpose of each exercise.

**Table 2:** The structured warm-up program used in the intervention group

**Exercise**	**Instructions**	Repetitions or duration*
1. One-legged knee squat	Slow movement with a smooth turn. Keep the pelvis in a horizontal position. Non-supporting foot held in front of the body with slightly flexed hip and knee.	
Level A	Hands on the hips.	3 × 8–15
Level B	Hold a ball over the head with straight arms.	3 × 8–15
Level C	Hands on the hips. Mark with the non-supporting foot just above the ground at the 12-02-04-06 o'clock positions.	3 × 5
Level D	Bend down while holding a ball and let the ball touch the ground outside the supporting foot. Make a diagonal movement upwards and raise the ball over the head with straight arms on the contralateral side.	3 × 8–15
Pair-exercise	Teammate stands slightly oblique in front of you. Hands on the hips and press a ball between the lateral sides of the feet of the non-supporting legs.	3 × 5–10
2. Pelvic lift	Supine position. Lift the pelvis from the ground while keeping the back straight.	
Level A	Both feet on the ground and hands across the chest.	3 × 8–15
Level B	One foot on the ground and the contralateral leg flexed in the hip and knee approximately 90 degrees with both hands on the knee.	3 × 8–15
Level C	One foot on a football and the contralateral leg flexed in the hip and knee approximately 90 degrees with the arms on the ground alongside the body for support.	3 × 8–15
Level D	One foot on the ground and the other in the air. Keep the upper arms on the ground for support with the elbows flexed 90 degrees. Push away the supporting foot and land on the contralateral foot.	3 × 8–15
Pair-exercise	Teammate stands with flexed knees and supports the heel of one of your feet in her hands. Hands across the chest and lift the pelvis.	3 × 8–15
3. Two-legged knee squat	Slow movement with a smooth turn. Keep the back in a straight position. Feet shoulder-wide apart with the soles in contact with the ground at all times during the squat.	
Level A	Hold a ball in front of the body with straight arms.	3 × 8–15
Level B	Hands on the hips.	3 × 8–15
Level C	Hold a ball over the head with straight arms.	3 × 8–15
Level D	Same as Level C, but continue the movement and rise up on the toes after returning to the starting position. Stay briefly in that position with good control.	3 × 8–15
Pair-exercise	Teammate stands next to you approximately 1 meter away, face opposite directions. Hold a ball between you with one hand and the other hand on the hip. Apply slight pressure on the ball while performing the knee squat.	3 × 8–15
4. The bench	Lift the body and keep it in a straight line.	
Level A	Prone position. Support on the knees and on the lower arms with the elbows kept under the shoulders.	15–30 seconds
Level B	Same as Level A but with support on the tip of the feet.	15–30 seconds
Level C	Same as Level B, but move the foot to the side and back to the starting position. Alternate sides.	15–30 seconds
Level D	Lie sideways with support on the foot and the lower arm with the elbow kept under the shoulder and the other hand on the hip. Lift the hip off the ground and stay briefly in that position with good control before slowly returning to the starting position.	3 × 5–10
Pair-exercise	Teammate stands behind you and holds your feet or lower legs. Lift the body and walk forward by using the hands on the ground.	15–30 seconds
5. The lunge	Take a step with a marked knee lift and a soft landing.	
Level A	Hands on the hips. Move forward with each step.	3 × 8–15
Level B	Hold a ball in front of the body with straight arms. Rotate the upper body while stepping forward and position the ball laterally of the front leg. Move forward with each step and alternate sides.	3 × 8–15
Level C	Hold a ball over the head with straight arms. Perform a forward lunge and push back with the front leg and return to the starting position.	3 × 8–15
Level D	Hold a ball in front of the body with straight arms. Perform a sideway lunge and return to the starting position.	3 × 8–15
Pair-exercise	Teammate stands in front of you 5–10 meters away. Perform a forward lunge while making a throw-in with a ball.	3 × 8–15
6. Jump/landing	Make a jump with a soft landing. Stay briefly in the landing position.	
Level A	Stand on one leg with the knee slightly bent and hands on the hips. Make a short forward jump and land on the same foot. Jump backwards to the starting position.	3 × 8–15
Level B	Stand on two legs shoulder-wide apart with the hands on the back. Make a sideways jump and land on one foot. Alternate sides.	3 × 8–15
Level C	Take a few quick steps on the same spot and make a short jump straight forward landing on one foot.	3 × 5
Level D	Same as level C, but change direction and jump to one side (90 degrees turn). Alternate sides.	3 × 5
Pair-exercise	Teammate stands in front of you approximately 5 meters away. Make a two-legged jump while heading a football and land on two legs.	3 × 8–15

Exercises 1, 3, 5 and 6: Focus on knee over foot position

Exercises 2, 3, 4 and 5: Ensure abdominal and gluteal muscle contraction for increased lower back support

* Number of repetitions unless otherwise specified.

The clubs assigned to the intervention group were instructed to perform the exercises during the warm-up at two training sessions per week throughout the entire 2009 competitive season. The exercises are preceded by 5 minutes of low-intensity running and take about 10–15 minutes to complete after familiarization. Coaches and players are encouraged to watch each other closely and give feedback about the execution of exercises. All players start on the first level of difficulty (A) and the coach clears each player's progress to the next level when she performs the exercise with good control, mainly focusing on core stability and proper knee alignment.

### Data collection

Data is collected during the competitive season from April until the end of the season in October (approximately 7 months). At the start of the study, a baseline form containing information about name, social security number, stature, body mass, menarche, family history of ACL injury, and the player's previous knee injuries and any current complaints is filled in by the player and parents/guardians. During the season, individual player exposure for each training session and match is reported by a coach or team leader on a computer-based registration form that is e-mailed to the research centre monthly. If a player has additional exposures outside the team, such as training or match play with a national team or with a senior team of the club, these are also registered on the exposure form. If a player sustains an acute knee injury during football training or match, the coach contacts the study therapist assigned to the club for evaluation of the injury. All acute knee injuries, except contusions, resulting in absence from play (time loss) are documented soon after the event by the study therapist on a standardized injury report form. If a severe knee injury is suspected, the player can be either advised to visit the nearest emergency department or referred to a study physician involved in the study. One physician experienced in diagnosing knee injuries is assigned to each regional district to assist the study therapists in the evaluation of severe knee injury. All players sustaining possible first-time ACL injuries (based on history and/or clinical examination) will be routinely examined with magnetic resonance imaging (MRI) by the study physicians to verify the diagnosis and to evaluate associated injuries. Suspected re-ruptures or secondary intraarticular injuries in knees with a previously reconstructed ACL will be assessed individually according to best practice. Injuries occurring at school, in other sports or during leisure time are not registered. Injuries are followed until full return to play or until 1 year post-injury.

### Compliance

At the start of the study, coaches in the intervention group deliver a preliminary training schedule specifying the two weekly sessions where the structured warm-up program is planned to be carried out. Any subsequent changes in the schedule need to be reported to the study therapist. The coach documents on the exposure form if the program has been carried out as scheduled. The study therapists will make unannounced visits to their teams in the intervention group twice during the study period (once during the spring season and once during the autumn season) to monitor compliance to the program. Study therapists are encouraged to contact teams with low compliance (fewer than 6 prevention sessions over a 4-week period) to motivate them and increase their compliance.

### Ethics

The study was approved by the Regional Ethical Review Board in Linköping (# M197-08). All players are verbally informed about the study by their coaches before the start of the study and also receive information to read together with their parents/guardians. Participation in the study is voluntary and all players sign written informed consent together with their parents/guardians according to the Declaration of Helsinki. None of the authors are involved in diagnosis or treatment of player injuries.

### Sample size

The sample size was based on previous data showing an average annual/seasonal incidence of ACL injury among female adolescent football players of 1.15% as reported in one prospective study [24] or in the control groups of previous knee injury prevention studies (Table 3) [14-17,19]. With a power of 80%, significance level of 5%, and an estimated risk reduction of 50% in the intervention group, randomization of approximately 4000 players to each group would be required [25].

**Table 3:** Annual/seasonal incidence of ACL injury in prospective cohort or controlled intervention studies in adolescent female football used for the sample size calculation.

**Study**	**Population**	**Study design**	**Number of players in the control group**	**Number of players with ACL injury (%)**
Hewett et al., 1999 [15]	High school	Controlled trial	193	2 (1.04%)
Heidt et al., 2000 [14]	Age 14–18 years	Randomized controlled trial	258	8 (3.10%)
Powell & Barber-Foss, 2000 [24]	High school	Prospective cohort study	6 642	33 (0.50%)
Mandelbaum et al., 2005 [16]	Age 14–18 years	Controlled trial	3 818	67 (1.76%)
Pfeiffer et al. 2006 [17]	High school	Controlled trial	189	0
Steffen et al., 2008 [19]	Age 13–17 years	Randomized controlled trial	947	5 (0.53%)

ACL: Anterior Cruciate Ligament

### Statistical analysis

The incidence will be calculated as number of injuries per 1000 playing hours. The primary outcome of the study is the incidence of ACL injury. The secondary outcomes are the incidence of any acute knee injury (except contusion) and the incidence of severe knee injury (defined as injury resulting in absence of more than 4 weeks). All outcomes will be evaluated according to the mechanism of injury (contact or non-contact). The exposure and injuries for a team in the intervention group is calculated from the first interventional session performed during the season (matched with the same date to a team in the control group). All analyses will be performed both according to the intention-to-treat principle and according to per protocol. The analyses will be performed with group identity concealed. Clustering will be accounted for in the analyses and the intracluster correlation coefficient will be computed. A Cox regression model with cluster as a random effect will be performed as well as a multilevel mixed model analysis based on Poisson regression. The relative risk reduction (RRR) is calculated using the rate in the control group divided by the rate in the intervention group and will be reported as a ratio with 95% confidence interval. Covariates in the analyses will include age, menarche, previous knee injury, match frequency and match play with other teams than the player's own. The absolute risk reduction (ARR) is calculated using the rate in the control group minus the rate in the intervention group. The number needed to treat (NNT) will be calculated based on the ARR. All statistical tests will be 2-sided and a significance level of < 0.05 will be used.

## Discussion

Knee injuries in football are common regardless of age, gender or playing level, but adolescent females seem to have the highest risk. Prevention of knee injury would be beneficial for players, clubs, insurance companies, and society. This cluster randomized controlled trial is designed to evaluate the effect of a commercially available structured warm-up program in adolescent female football. The strengths of our study include the large study sample compared to most of previous research in the field [13-15,17-20] and the use of a recently validated methodology [22,23]. In addition, the documentation of background information and individual player exposure will allow us to evaluate the risk of knee injury and control for possible interaction or confounding factors.

One limitation of the present trial is that it was not possible to perform a power calculation for a cluster RCT from exposure-based incidence rates of ACL injury, since valid data is lacking in the literature. Consequently, we cannot know for sure that the study sample is large enough. Another limitation is that the sports physical therapists supporting the teams and evaluating knee injuries are not blinded to group allocation. We decided to favour a professional evaluation of players' acute knee injuries by the physical therapists and it was not possible to recruit additional therapists specifically to assist teams with data collection and to instruct and monitor compliance to the preventive program. Due to the nature of the intervention, blinding of players or coaches to group allocation is not possible. Study physicians evaluating severe knee injuries are, however, blinded to group allocation.

## Competing interests

The authors declare that they have no competing interests.

## Authors' contributions

MH and MW designed the study and are the principle researchers responsible for recruitment and data collection. IA contributed to the design of the study and performed the randomization. MH and MW are study guarantors. All authors contributed to the writing of the protocol and approved the final manuscript.

## Pre-publication history

The pre-publication history for this paper can be accessed here:


